# A Survey of New Temperature-Sensitive, Embryonic-Lethal Mutations in *C. elegans*: 24 Alleles of Thirteen Genes

**DOI:** 10.1371/journal.pone.0016644

**Published:** 2011-03-01

**Authors:** Sean M. O'Rourke, Clayton Carter, Luke Carter, Sara N. Christensen, Minh P. Jones, Bruce Nash, Meredith H. Price, Douglas W. Turnbull, Aleena R. Garner, Danielle R. Hamill, Valerie R. Osterberg, Rebecca Lyczak, Erin E. Madison, Michael H. Nguyen, Nathan A. Sandberg, Noushin Sedghi, John H. Willis, John Yochem, Eric A. Johnson, Bruce Bowerman

**Affiliations:** The Institute of Molecular Biology, University of Oregon, Eugene, Oregon, United States of America; Brown University, United States of America

## Abstract

To study essential maternal gene requirements in the early *C. elegans* embryo, we have screened for temperature-sensitive, embryonic lethal mutations in an effort to bypass essential zygotic requirements for such genes during larval and adult germline development. With conditional alleles, multiple essential requirements can be examined by shifting at different times from the permissive temperature of 15°C to the restrictive temperature of 26°C. Here we describe 24 conditional mutations that affect 13 different loci and report the identity of the gene mutations responsible for the conditional lethality in 22 of the mutants. All but four are mis-sense mutations, with two mutations affecting splice sites, another creating an in-frame deletion, and one creating a premature stop codon. Almost all of the mis-sense mutations affect residues conserved in orthologs, and thus may be useful for engineering conditional mutations in other organisms. We find that 62% of the mutants display additional phenotypes when shifted to the restrictive temperature as L1 larvae, in addition to causing embryonic lethality after L4 upshifts. Remarkably, we also found that 13 out of the 24 mutations appear to be fast-acting, making them particularly useful for careful dissection of multiple essential requirements. Our findings highlight the value of *C. elegans* for identifying useful temperature-sensitive mutations in essential genes, and provide new insights into the requirements for some of the affected loci.

## Introduction

To investigate essential gene requirements in model organisms, multiple approaches have been used to reduce gene function and infer gene requirements based on the resulting mutant phenotypes. Non-conditional mutations that inactivate genes can be used to study essential requirements, but such mutations must be maintained in heterozygotes and homozygous mutant progeny identified among progeny that vary in genotype. Furthermore, one gene can have multiple essential requirements during an organism life cycle, precluding investigation of all but the first essential requirement in progeny homozygous for a non-conditional mutation. To bypass early essential requirements in multicellular organisms, mitotic recombination [1], [2], cell transplantation [3], [4], or loss of extrachromosomal arrays [5] can be used to generate clones of homozygous mutant cells within otherwise heterozygous or wild-type individuals. But even within mutant clones of cells, only a single, early essential requirement can be examined, and the degree of control over the place and timing of mutant clone generation can vary substantially. Weak alleles of essential genes can sometimes bypass early essential requirements to permit the study of later requirements, and both RNA interference (RNAi) and small molecule inhibitors can in some cases be used to reduce gene function at multiple times during the life an organism [6], [7]. However, the small molecule inhibitors suffer in some cases from a lack of gene specificity, a lack of penetrance in reducing gene function, or reduced bioavailability to the targeted protein. Thus, both small molecule inhibitors and RNAi remain limited in scope with respect to their use in many multicellular organisms. Finally, for genes that are expressed both maternally and zygotically, maternal expression of a wild-type allele can in some cases compensate for lack of zygotic expression in homozygous mutant progeny, precluding the identification of some gene requirements early in development when non-conditional alleles result in lethality due to later essential zygotic requirements.

When available, fast-acting temperature-sensitive (TS) gene mutations are perhaps the most powerful tool for dissecting multiple requirements for essential genes. While some conditional mutations are cold-sensitive (inactivating a gene product only at low temperatures), most conditional mutations are heat-sensitive (inactivating gene products only at high temperatures). TS mutations can also be either fast or slow acting, with fast-acting mutations causing amino acid changes that presumably destabilize a protein such that it unfolds or adopts a non-functional structure shortly after up-shifting the organism to a restrictive temperature. Slow acting mutations presumably remain active at all temperatures when made at the permissive temperature, and must be replaced by newly synthesized, inactive protein after up-shifting to the restrictive temperature. Particularly with fast-acting TS mutations, one can identify multiple essential requirements, and define temperature-sensitive periods of gene requirements, sometimes even within a single cell cycle, or during the entire life span of an organism, by performing temperature up-shifts and down-shifts at different times [8], [9]. Conditional mutations are also useful in that they allow for the easy propagation of homozygous mutant strains at the permissive temperature, and can be used to sensitize genetic backgrounds at intermediate temperatures for use in screens designed to identify second-site modifier loci as enhancers or suppressors of viability [10], [11], [12]. Moreover, site-directed mutagenesis can be used to engineer TS amino acid alterations in orthologous genes in other organisms. For example, a TS mutation in *C. elegans* dynein heavy chain, *dhc-1*, was engineered in the *S. cerevisiae* ortholog and was found to confer TS function [8]. In another case a ts allele of *src* was engineered in the *D. melanogaster* gene *sevenless* [10]. While TS mutations may not be useful for in vivo studies with mammalian model systems, some TS alleles have been identified in mammalian cell culture [13].

Not surprisingly, TS mutations isolated by mutagenizing populations of an organism are rare relative to non-conditional loss-of-function mutations. Many mutations can partially or fully inactivate a gene: for example, single nucleotide mutations can introduce early stop codons at one of many possible sites in most open reading frames. In contrast, relatively few mutations perturb protein function such that the outcome is conditional. For example, TS mutations often involve amino acid substitutions (mis-sense mutations) within the hydrophobic core of a folded protein that destabilize protein folding at higher temperatures [14].

Because TS mutations are relatively rare, they have been used most extensively in model organisms that are amenable to screens that enable one to search through large populations of mutagenized individuals for relatively rare conditional mutants. For example, TS mutants have been used extensively in budding yeast and fission yeast to identify essential gene functions [15], [16], including many cell division cycle (CDC) genes that were discovered and characterized in both of these yeasts by screening for TS CDC mutant strains [17], [18]. Shifting CDC mutant yeast to restrictive temperatures resulted in specific cell cycle arrest that elegantly revealed when the gene product was required [19]. TS mutants have also been utilized in *Drosophila melanogaster* [20], although far fewer examples exist and most have been identified fortuitously. Mammalian cell lines also have been used to isolate TS alleles of essential genes [13], but again relatively few examples exist.

TS mutations are now being used more and more extensively to probe gene function in the nematode *Caenorhabditis elegans*. Indeed, this organism is largely unique in being an animal model in which one can with relative ease identify rare conditional mutations in essential genes. Since the initial establishment of this nematode as a model organism, screening for conditional *C. elegans* mutants has been more feasible than in other animals, in part because it is self-fertile [21], [22], [23]. More recently, the innovation of using of egg-laying defective strains made *C. elegans* a powerful system for isolating non-conditional mutation in essential genes required for embryogenesis [24]. Modifications to the screening procedures that use egg-laying defective strains subsequently made it possible to isolate with relative ease thousands of conditional mutations in essential genes [25], [26], [27].

While one can efficiently isolate conditional, embryonic-lethal *C. elegans* mutants, positional cloning of the mutant loci has remained laborious and time consuming, substantially limiting the utility of mutant screens, particularly given how readily one can use RNA interference to probe essential *C. elegans* gene functions [6], [28], [29], [30], [31], [32]. However, the advent of next generation DNA sequencing technology is now making it possible to identify much more rapidly the genes affected in mutant strains [33].

Here we report our identification of 24 conditional mutants in thirteen different essential *C. elegans* loci. To further promote the use and isolation of conditional mutations in essential *C. elegans* genes, we have surveyed this collection of new conditional mutants for essential gene requirements during both larval and early embryonic development, and we have determined whether all are fast or slow acting. We also report the mutations responsible for conditional lethality for most of these alleles, and whether the affected residues are conserved in other organisms.

## Results

Over the past several years, using chemical mutagenesis of egg-laying defective *lin-2(-)* mutants with either ethyl methanesulfonate or ethyl nitrosourea, we have isolated conditional mutations in multiple essential *C. elegans* genes that already had been characterized using either mutant alleles or RNAi to reduce gene function. Here we report our identification of 24 conditional mutations in thirteen different essential genes, and an analysis of the conditional nature of the mutations. Most of these mutations were mapped with traditional methods, using both visible markers and individually amplified Single Nucleotide Polymorphisms (SNPs) to score meiotic recombination events. The affected loci were then identified using both complementation tests with previously identified alleles, and DNA sequencing of candidate genes in regions to which the mutations were mapped. More recently, we have begun to take advantage of next generation Illumina DNA sequencing based methods to greatly accelerate the pace at which we can identify the affected genes in mutants isolated after mutagenesis of nematode populations. In the following sections, we describe the conditional mutations we have characterized for each affected locus.

### α- and β-Tubulin Mutations

**Figure 1:**
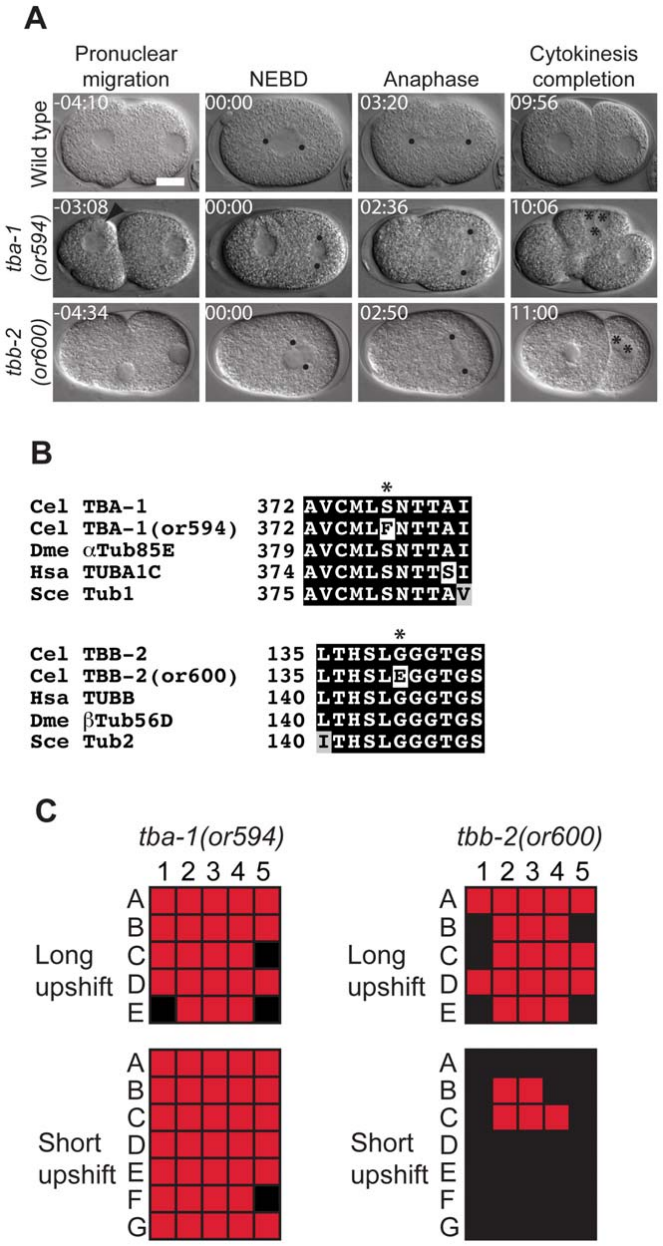
*tba-1* and *tbb-2* tubulin mutants. A. Differential interference contrast (DIC) time-lapse images of wild-type, *tba-1(or594 *ts*),* and *tbb-2(or600 *ts*)* embryos. In the two tubulin mutants the P_0_ spindle often is positioned transverse to the anterior posterior axis, and daughter cells contained multiple nuclei. The *tba-1(or594 *ts*)* embryo was from a∼1 min. upshift and the *tbb-2(or600 *ts*)* embryo was shifted to the restrictive temperature for 8 hours. Black dots represent centrosomes/spindle poles, asterisks denote multiple nuclei per cell, and the arrowhead indicates a second maternal pronucleus. Times in min:sec are given relative to nuclear envelope breakdown (NEBD). Scale bar, 10 µm. B. Amino acid alterations in the two mutants. Asterisks indicates the changed residues. Homologous proteins are aligned below the *C. elegans* protein. C. Defect maps for individual embryos observed during time-lapse recordings; embryos are listed on the left and phenotypes are listed on the top: 1; pronuclei meet prior to NEBD, 2; Nuclear centrosomal complex centration, 3; Nuclear centrosomal complex rotation, 4; spindle alignment, 5; one nucleus per cell at two cell stage. In the long upshifts, hermaphrodites were transferred to the restrictive temperature for 5–8 hours. In the short upshifts, embryos were harvested from hermaphrodites grown at 15°C and immediately mounted on agar pads for imaging, which took about 1 minute. Red color indicates a defective trait, black color represents the lack of a defect.

Microtubules are polymers of *α*- and *β*-tubulin and are essential for multiple cellular activities, including meiotic and mitotic spindle function. In *C. elegans* embryos there are two functionally redundant *α*-tubulin genes, *tba-1* and *tba-2*, and also two functionally redundant *β*-tubulin genes, *tbb-1* and *tbb-2*. While reducing the function of any one gene with RNAi does not result in penetrant phenotypes, reducing the function of either gene pair simultaneously with RNAi results in severe meiotic and mitotic spindle defects and embryonic lethality [34], [35]. In addition, we have previously identified conditional, semi-dominant mutations in *tba-1* and *tbb-2* that appear to destabilize microtubules and cause highly penetrant embryonic lethality when adult worms are raised at the restrictive temperature of 26 °C. We have now identified one new *tba-1* mutant, *or594 *sd,ts, and one new *tbb-2* allele, *or600 *sd,ts. Each of the alleles is semi-dominant (Table 1), as expected given the redundancy of the two gene pairs. We used genetic crosses to place the *or594 *ts and *or600 *ts alleles in trans to the previously identified alleles *tba-1(or346 *ts*)* and *tbb-2*(*or362 *ts*)*, respectively. The progeny of both *or594 *ts*/tba-1(or346 *ts*)* and *or600 *ts*/tbb-2(or362 *ts*)* worms exhibited fully penetrant embryonic lethality (data not shown). This is in contrast to the partially penetrant embryonic lethality observed when any of these alleles were in trans to a wild-type copy of the corresponding gene (Table 1; [34], [35]), consistent with our conclusion that *or594 *ts and *or600 *ts are *tba-1* and *tbb-2* alleles, respectively. As shown in Figure 1, we see penetrant defects in embryos produced by homozygous *tba-1(or594 *ts*)* and by homozygous *tbb-2(or600 *ts*)* mutant worms raised at 26°C from the L4 stage to adulthood (hereafter called mutant embryos). As reported for other conditional and semi-dominant mutations in *tba-1* and *tbb-2*, we observed defects in meiotic spindle function, pronuclear migration, nuclear centrosomal complex (NCC) centration and rotation, mitotic spindle positioning and size, chromosome segregation and cytokinesis during the first mitotic cell cycle (Fig. 1A). Although the new *tba-1(or594 *sd,ts*)* mutant and a previous allele, *tba-1(or346 *sd,ts*)*, were isolated in different screens, the mutations are identical and change the highly conserved serine at position 377 to phenylalanine (Fig. 1B, Table 2). The mutation in *tbb-2(or600 *sd,ts*)* changes a highly conserved glycine at amino acid 140 to a glutamic acid (Fig. 1B, Table 2).The phenotypes were similar to other dominant tubulin alleles but are not as severe as RNAi-mediated co-depletion of either gene pair, and thus represent an approach to disrupt microtubule function less severely than co-depleting either of the gene pairs [34], [35]. The *tba-1(or594 *sd,ts*)* mutant showed highly penetrant defects when shifted to the restrictive temperature for long (5–8 hours) or short (∼1 minute) upshifts, while the *tbb-2(or600 *sd,ts*)* mutant showed penetrant defects only after long upshifts (Fig. 1C and Table 3). Shifting *tba-1(or594 *sd,ts*)* mutants to the restrictive temperature at the L1 larval stage resulted in mostly fertile worms but about 20% were sterile, while similar shifts with *tbb-2(or600 *sd,ts*)* mutants resulted in adult worms that were fertile but produced small broods (Table 4).

**Table 1:** Embryonic lethality of the TS mutants.

Gene	Allele	Homozygote Embryonic Viability (15°C)	Homozygote Embryonic Viability (26°C)	Heterozygote Embryonic Viability (26°C)
*dnc-1*	*or404*	98.6%, *n* = 435	0.0%, *n* = 226	99.3%, *n* = 365
*dnc-1*	*or676*	18.7%, *n* = 626	1.62%, *n* = 747	88.6%, *n* = 474
*dnc-4*	*or618*	92.7%, *n* = 236	12.7%, *n* = 259	97.7%, *n* = 342
*dnc-4*	*or633*	99.4%, *n* = 486	3.15%, *n* = 444	77.0%, *n* = 364
*lit-1*	*or393*	95.6%, *n* = 471	1.55%, *n* = 554	99.1%, *n* = 374
*mei-1*	*or642*	97.3%, *n* = 1013	0.25%, *n* = 1329	98.5%, *n* = 506
*mei-1*	*or646*	78.8%, *n* = 438	0.40%, *n* = 1135	98.5%, *n* = 613
*mex-1*	*or286*	53.1%, *n* = 675	3.4%, *n* = 1327	93.1%, *n* = 249
*par-2*	*or373*	99.6%, *n* = 282	2.0%, *n* = 251	not tested
*par-2*	*or539*	94.8%, *n* = 194	42.1%, *n* = 554	97.0%, *n* = 371
*par-2*	*or640*	98.7%, *n* = 230	0.0%, *n* = 318	89.7%, *n* = 226
*plk-1*	*or683*	62.3%, *n* = 630	0.80%, *n* = 424	97.8%, *n* = 383
*rsa-1*	*or598*	99.8%, *n* = 444	1.66%, *n* = 543	100%, *n* = 766
*spd-2*	*or293*	87.8%, *n* = 245	0.0%, *n* = 341	99.7%, *n* = 378
*spd-2*	*or493*	95.6%, *n* = 298	1.51%, *n* = 265	100%, *n* = 603
*spd-2*	*or454*	99.0%, *n* = 412	30.0%, *n* = 410	99.3%, *n* = 412
*spd-2*	*or655*	58.3%, *n* = 518	0.26%, *n* = 388	98.8%, *n* = 345
*sur-6*	*or550*	72.8%, *n* = 556	16.0%, *n* = 362	96.1%, *n* = 385
*tba-1*	*or594*	88.5%, *n* = 278	0.00%, *n* = 402	83.9%, *n* = 261
*tbb-2*	*or600*	99.1%, *n* = 551	0.81%, *n* = 745	58.4%, *n* = 294
*zyg-1*	*or278*	78.6%, *n* = 1091	0.0%, *n* = 685	98.7%, *n* = 236
*zyg-1*	*or297*	93.8%, *n* = 697	0.38%, *n* = 529	99.6%, *n* = 275
*zyg-1*	*or409*	99.5%, *n* = 411	0.0%, *n* = 396	96.1%, *n* = 233
*zyg-1*	*or1018*	97.8%, *n* = 276	0.0%, *n* = 407	96.3%, *n* = 246

**Table 2:** Sequence alterations in the TS mutants.

Gene	Allele	Transcript^1^	Codon(s) mutated^1^	Amino acid change^1^	Nucleotide change^1^	Transcript nucleotide (spl/unspl)^1^^,^ 2
*dnc-1*	*or404*	ZK593.5	1237	R>C	C>T	3709 (spl)
*dnc-1*	*or676* 3	ZK593.5	452	L>P	T>C	1355 (spl)
*dnc-1*	*or676* 3	ZK593.5	1247	V>L	G>C	3739 (spl)
*dnc-4*	*or618*	C26B2.1	359	V>G	T>G	1076 (spl)
*dnc-4*	*or633*	C26B2.1	-	-	G>A	604 (unspl)
*lit-1*	*or393*	W06F12.1a	331	I>F	A>T	991 (spl)
*mei-1*	*or642*	T01G9.5a.1	202	K>Q	A>C	604 (spl)
*mei-1*	*or646*	T01G9.5a.1	202	K>Q	A>C	604 (spl)
*mex-1*	*or286*	W03C9.7.1	13	Q>STOP	C>T	37 (spl)
*plk-1*	*or683*	C14B9.4a.1	547	M>K	T>A	1640 (spl)
*rsa-1*	*or598*	C25A1.9a	319	D>G	A>G	956 (spl)
*spd-2*	*or293*	F32H2.3.1	573	G>S	G>A	1717 (spl)
*spd-2*	*or493*	F32H2.3.1	551	R>H	G>A	1652 (spl)
*spd-2*	*or454*	F32H2.3.1	-	-	G>A	2175 (unspl)
*spd-2*	*or655*	F32H2.3.1	189–270	Deletion	Deletion	565–810Δ spl)
*sur-6*	*or550*	F26E4.1	140	W>R	T>C	418 (spl)
*tba-1*	*or594*	F26E4.8.1	377	S>F	C>T	1130 (spl)
*tbb-2*	*or600*	C36E8.5.1	140	G>E	G>A	419 (spl)
*zyg-1*	*or278*	F59E12.2.1	354	P>S	C>T	1060 (spl)
*zyg-1*	*or297*	F59E12.2.1	652	D>N	G>A	1954 (spl)
*zyg-1*	*or409*	F59E12.2.1	670	D>N	G>A	2008 (spl)
*zyg-1*	*or1018*	F59E12.2.1	498	V>A	T>C	1493 (spl)
*par-2*	*or373*	F58B6.3a	71	C>Y	G>A	212 (spl)

1 Transcripts, positions, and sequences are from the WS210 referential release of Wormbase.

2 Positions are provided for either spiced (spl) or unspliced (unspl) transcripts.

3 *dnc-1(or676)* was a double mutant.

**Table 3:** Determination if the TS mutations are potentially fast-acting.

Gene	Allele	Potentially Fast-acting^1^
*dnc-1*	*or404*	Yes
*dnc-1*	*or676*	Unclear^3^
*dnc-4*	*or618*	Yes
*dnc-4*	*or633*	Yes
*lit-1*	*or393*	Not tested
*mei-1* 2	*or642*	Yes
*mei-1* 2	*or646*	Yes
*mex-1*	*or286*	Not tested
*par-2* 2	*or373*	Yes
*par-2* 2	*or539*	Yes
*par-2* 2	*or640*	Yes
*plk-1*	*or683*	Unclear^3^
*rsa-1*	*or598*	Yes
*spd-2*	*or293*	Yes
*spd-2*	*or493*	Unclear^4^
*spd-2*	*or454*	Unclear^4^
*spd-2*	*or655*	Unclear^3^
*sur-6*	*or550*	Unclear^3^
*tba-1*	*or594*	Yes
*tbb-2*	*or600*	No
*zyg-1* 2	*or278*	No
*zyg-1* 2	*or297*	Yes
*zyg-1* 2	*or409*	Yes
*zyg-1* 2	*or1018*	No

1 We determined if an allele was potentially fast-acting in the following manner: We mounted embryos produced at 15°C on microscope slides and immediately made time-lapse videomicrographs at a room maintained at 24°C. If defects similar to those observed after long temperature shifts were found in at least 20% of the embryos and if there was little embryonic lethality at 15°C, we conclude that the allele may be fast-acting. We have labeled these cases as “Yes”. However, if there was significant embryonic lethality at 15°C, we cannot conclude that the presence of cellular defects after short upshifts is due to the upshift or to defects that occur even at 15°C. We have labeled these cases as “Unclear”.

2 For *mei-1, par-2,* and *zyg-1*, we incubated mutant worms at 26°C for 30 minutes prior to imaging (instead of the usual∼1 min. upshift) because the gene products appeared to be required prior to when we started our imaging (pronuclear migration).

3 High lethality at the permissive temperature precludes making a determination.

4 The low penetrance of severe defects precludes making a determination.

**Table 4:** The phenotypes of the TS mutants when grown at the restrictive temperature from the L1 larval stage.

Gene	Allele	L1 upshift phenotype^1^
*dnc-1*	*or404*	Egl/Emb
*dnc-1*	*or676*	Ste/Sb/Emb
*dnc-4*	*or618*	Egl/Emb
*dnc-4*	*or633*	Sb/Emb
*lit-1*	*or393*	Emb
*mei-1*	*or642*	Emb
*mei-1*	*or646*	Emb
*mex-1*	*or286*	Ste/Sb/Emb
*par-2*	*or373*	Emb
*par-2*	*or539*	Emb
*par-2*	*or640*	Ste
*plk-1*	*or683*	Ste/Pvl
*rsa-1*	*or598*	Ste
*spd-2*	*or293*	Ste/Pvl
*spd-2*	*or493*	Sb/Emb
*spd-2*	*or454*	Emb
*spd-2*	*or655*	Emb
*sur-6*	*or550*	Emb
*tba-1*	*or594*	Emb/Ste
*tbb-2*	*or600*	Sb/Emb
*zyg-1*	*or278*	Sb/Emb
*zyg-1*	*or297*	Emb
*zyg-1*	*or409*	Sb/Emb
*zyg-1*	*or1018*	Sb/Emb

1 We determined the L1 upshift phenotype by plating hypochlorite-synchronized L1 larvae and incubating them at 26°C until they reached adulthood. Abbreviations: Egl: egg laying-defective, Emb: embryonic lethal, Pvl: protruding vulva, Sb: small broods, Ste: sterile.

### Protein phosphatase 2A mutants

**Figure 2:**
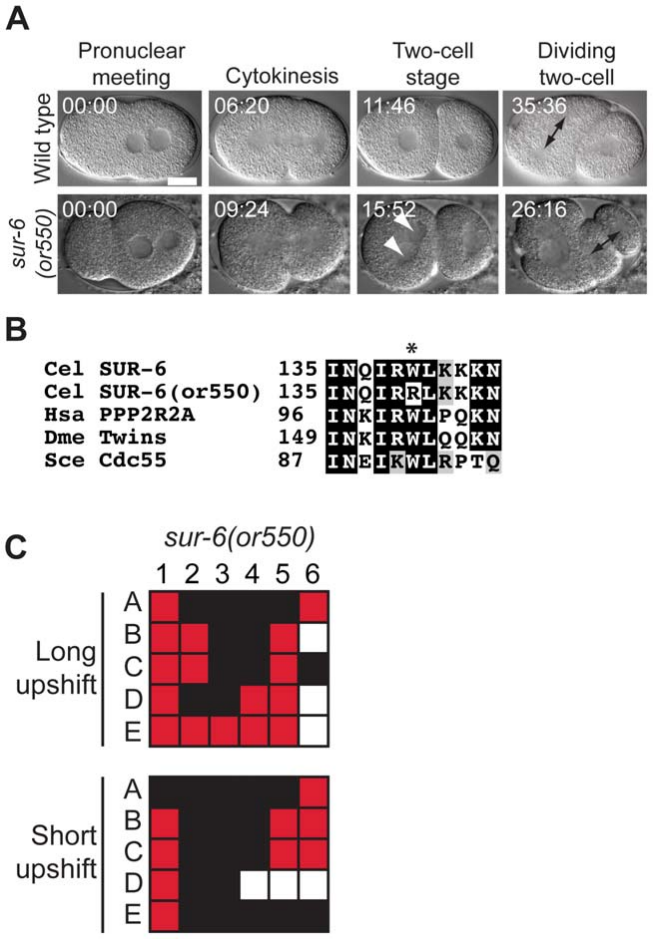
A *sur-6* mutant. A. DIC time-lapse images of wild-type and *sur-6(or550 *ts*)* embryos. In the *sur-6* mutant the male pronucleus is small, the AB cell contains two nuclei, and the P_1_ cell begins mitosis before the AB cell. The *sur-6(or550 *ts*)* embryo was shifted to the restrictive temperature for ∼1 min. prior to imaging. White arrowheads denote multiple nuclei per cell, and the arrows in the last panels indicate the first mitotic spindle at the two cell stage. Times in min:sec are given relative to pronuclear meeting. Scale bar, 10 µm. B. Amino acid alteration in the mutant. Asterisk indicates the changed residue. Homologous proteins are aligned below the *C. elegans* protein. C. Defect maps for individual embryos observed during time-lapse recordings. In this and all subsequent figures, embryos are listed on the left and phenotypes are listed on the top: 1; Male pronuleus normal size, 2; Nuclear centrosomal complex centration, 3; spindle alignment, 4; successful cytokinesis, 5; one nucleus per cell at two cell stage, 6; AB divides first at two cell stage. In the long upshifts, hermaphrodites were transferred to the restrictive temperature for 5–8 hours. In the short upshifts, embryos were harvested from hermaphrodites grown at 15°C and immediately mounted on agar pads for imaging, which took ∼1 min. In this and all subsequent figures, red color indicates a defective trait, black color represents the lack of a defect, and white indicates that the trait was not visible in the recording.

Protein phosphatase 2A is composed of a catalytic subunit and regulatory subunits known as B, B′, and B′′. The regulatory subunits provide targeting specificity thereby linking the catalytic subunit to various protein substrates throughout the cell cycle. SUR-6 is the B′ subunit in *C. elegans* and has known functions during embryonic and vulval development [36], [37]. We have identified a recessive conditional mutation in *sur-6, or550 *ts. The *sur-6(or550 *ts*)* mutant embryos produced after shifting homozygous L4 larvae to the restrictive temperature exhibited small male pronuclei, defects in NCC centration, and chromosome segregation defects during mitosis in the one-cell embryo (called P_0_), and the posterior P_1_ cell in 2-cell stage embryos often divided before its anteriorly positioned sister, called AB (Fig. 2A, and as reported previously for a different allele of *sur-6*, (*sv30*) [36]). In genetic crosses, *sur-6(or550 *ts*)* failed to complement *sur-6(sv30)* (data not shown). The amino acid alteration in *sur-6(or550 *ts*)* changes a highly conserved tryptophan to arginine at position 140 (Fig. 2B). We could not determine if the allele was fast-acting, even though some phenotypes were observed after short upshifts (Fig. 2C), because of significant embryonic lethality at the permissive temperature (Table 1).

**Figure 3:**
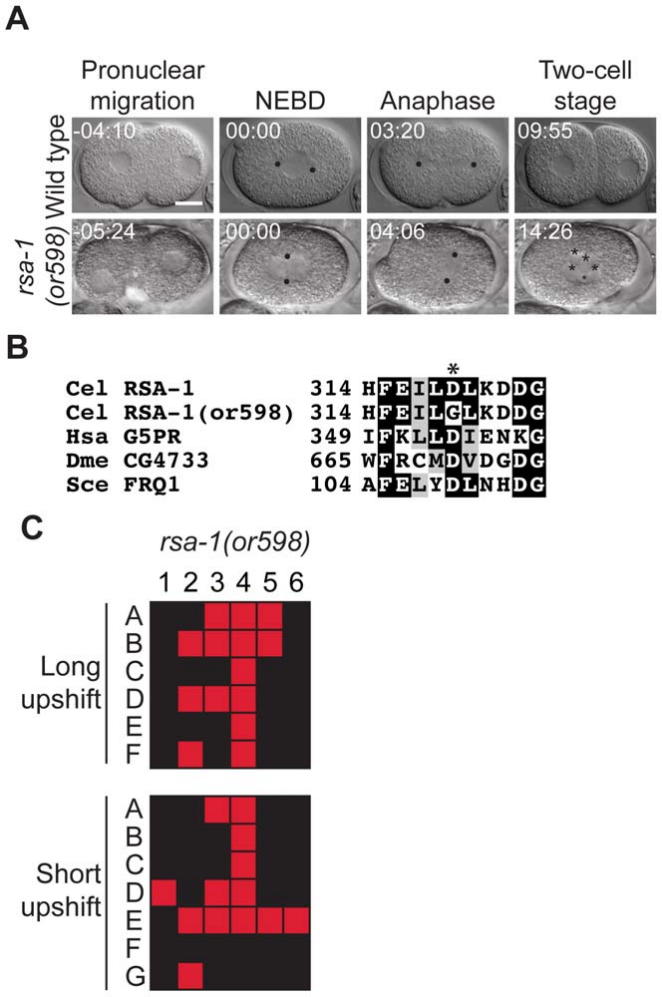
An *rsa-1* mutant. A. DIC time-lapse images of wild-type and *rsa-1(or598 *ts*)* embryos. In the *rsa-1* mutant the nuclear centrosomal complex (NCC) failed to rotate and a small transverse P_0_ spindle assembled, cytokinesis failed, and multiple nuclei were present at the two cell equivilent stage. The *rsa-1(or598 *ts*)* embryo was shifted to the restrictive temperature for∼1 min. prior to imaging. Black dots represent centrosomes/ spindle poles and asterisks denote multiple nuclei per cell at the two cell stage. Times in min:sec are given relative to NEBD. Scale bar, 10 µm. B. Amino acid alteration in the mutant. Asterisk indicates the changed residue. Homologous proteins are aligned below the *C. elegans* protein. C. Defect map for individual embryos observed during time-lapse recordings: embryos are listed on the left and phenotypes are listed on the top: 1; pronuclei meet prior to NEBD, 2; Nuclear centrosomal complex centration, 3; Nuclear centrosomal complex rotation, 4; spindle size, 5; successful cytokinesis, 6; one nucleus per cell at two cell stage. In the long upshifts, hermaphrodites were transferred to the restrictive temperature for 5–8 hours. In the short upshifts, embryos were harvested from hermaphrodites grown at 15°C and immediately mounted on agar pads for imaging, which took ∼1 min.

The PP2A B′′ subunit is encoded by *rsa-1* in *C. elegans* [38]. We identified one new recessive allele of *rsa-1, or598 *ts. Like previously characterized alleles or *rsa-1(RNAi)* knockdown, *rsa-1(or598 *ts*)* mutant embryos showed multiple defects in the one-cell embryo including defective NCC centration and rotation, small spindles, and chromosome segregation defects (Fig. 3A). *rsa-1(or598 *ts*)* is the only TS allele for *rsa-1*, with a conserved aspartic acid changed to glycine at position 319 (Fig. 3B). The *rsa-1* mutant was fast-acting for many of the phenotypes (Fig. 3C and Table 3), and L1 larval upshift resulted in sterile adults (Table 4).

### Dynactin mutants

**Figure 4:**
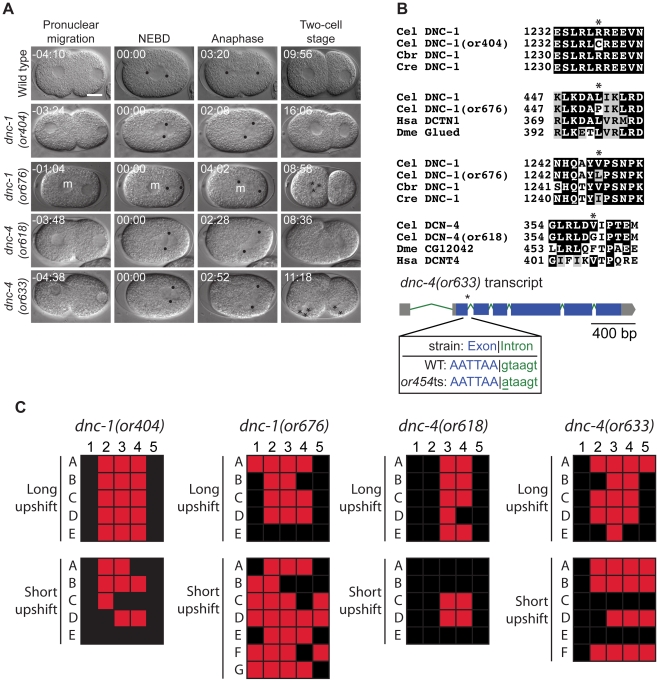
Dynactin mutants. A. DIC time-lapse images of wild-type, *dnc-1(or404 *ts*), dnc-1(or676 *ts*), dnc-4(or618 *ts*), and dnc-4(or633 *ts*)* embryos. In the dynactin mutants the NCC often failed to centrate and rotate, the P_0_ spindle was oriented transverse to the anterior posterior axis, and multiple nuclei were present per cell at the two cell stage. The *dnc-1(or404 *ts*)* embryo was obtained from a hermaphrodite shifted to the restrictive temperature for 8 hours, the *dnc-1(or676 *ts*)* and *dnc-4(or633 *ts*)* embryos were shifted to the restrictive temperature for ∼1 min. prior to imaging, and the *dnc-4(or618 *ts*)* embryo was obtained from a hermaphrodite shifted to the restrictive temperature for 7 hours. Black dots represent centrosomes/spindle poles, asterisks denote multiple nuclei per cell, and the “m” denotes the maternal pronucleus that did not meet the male pronucleus prior to NEBD. Times in min:sec are given relative to nuclear envelope breakdown (NEBD). Scale bar, 10 µm. B. Sequence alterations in the mutants. Asterisks indicate the changed residues (or nucleotide for *dnc-4(or633 *ts). Homologous proteins are aligned below the *C. elegans* proteins. *dnc-4(or633 *ts*)* contains a mutation in an intron that may affect RNA splicing. C. Individual embryos observed during time-lapse recordings: embryos are listed on the left and phenotypes are listed on the top: 1; pronuclei meet prior to NEBD, 2; Nuclear centrosomal complex centration, 3; Nuclear centrosomal complex rotation, 4; spindle alignment, 5 one nucleus per cell at two cell stage. In the long upshifts, hermaphrodites were transferred to the restrictive temperature for 5–8 hours. In the short upshifts, embryos were harvested from hermaphrodites grown at 15°C and immediately mounted on agar pads for imaging, which took ∼1 min.

Dynactin is a protein complex that simultaneously binds both microtubules and cytoplasmic dynein [39]. Because dynactin cross-links dynein and microtubules, it increases dynein motor processivity. We isolated two *dnc-1* alleles, *or404 *ts and *or676 *ts, and two new *dnc-4* alleles, *or618 *ts and *or633 *ts. All of the dynactin mutants show similar microtubule-related defects in one-cell embryos, as previously reported for *dnc-1* and *dnc-2* using RNAi depletion [40]. Instead of the nuclear-centrosomal complex (NCC) centering in the embryos after meeting, the NCC remains in the posterior in the dynactin mutants. In addition, the NCC fails to rotate causing the P_0_ spindle to assemble transverse to the anterior-posterior embryonic axis (Fig. 4A). *dnc-1(or404 *ts*)* displayed a somewhat weaker cellular phenotype than the other three *dnc* alleles (Fig. 4C) and, interestingly, *dnc-1(or676 *ts*)* and *dnc-4(or633 *ts*)* were either semi-dominant or haploinsufficient (Table 1), while the other two *dnc* alleles appeared to be recessive. In genetic crosses, *dnc-1(or404 *ts*)* failed to complement *dnc-1(or676 *ts*)*, and *dnc-4(or618 *ts*)* failed to complement *dnc-4(or633 *ts*)* (data not shown). Although *dnc-1(or404 *ts*)* was previously characterized [41], [42], we have more extensively documented the phenotypes here. No alleles of *dnc-4* have been previously reported nor has it been extensively characterized in *C. elegans*. The *dnc-1(or404 *ts*)* allele changes amino acid 1237 from an asparagine to a cysteine. *dnc-1(or676 *ts*)* has two changes: one at position 452 (leucine to proline) and the other at position 1247 (valine to leucine). *dnc-4(or618 *ts*)* substitutes valine for a glycine at position 359 and *dnc-4(or633 *ts*)* has an altered splice donor site after the first exon (G to A) at nucleotide 604 in the unspliced RNA molecule. The *dnc-1(or404 *ts*)* and *dnc-4(or633 *ts*)* mutants appeared to be fast-acting (Fig. 4C and Table 3). Finally, in tests where L1 larvae were raised to adulthood at the restrictive temperature, we found that *dnc-1(or404 *ts*)* and *dnc-4(or618 *ts*)* worms displayed egg-laying defects, *dnc-1(or676 *ts*)* worms were sterile or produced small numbers of progeny, while *dnc-4(or633 *ts*)* worms also produced reduced numbers of progeny (Table 4).

### *mei-1/*Katanin mutants

**Figure 5:**
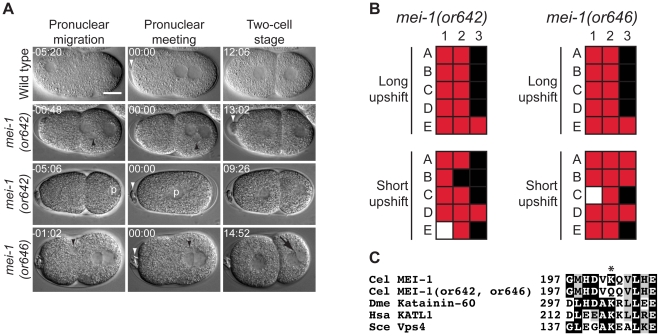
*mei-1* mutants. A. DIC time-lapse images of wild-type, *mei-1(or642 *ts*) and mei-1(or646 *ts*)* embryos. In the *mei-1* mutants the polar bodies were large and misshapen and embryos contained multiple [top *mei-1(or642 *ts*)* embryo and *mei-1(or646 *ts*)*] or zero maternal pronuclei (second *mei-1(or642 *ts*)* embryo). The two *mei-1(or642 *ts*)* embryos were obtained from a hermaphrodite shifted to the restrictive temperature for 30 minutes, the *mei-1(or646 *ts*)* embryo was obtained from a hermaphrodite shifted to the restrictive temperature for 7 hours prior to imaging. White arrowheads indicates polar bodies, black arrowheads indicate multiple maternal pronuclei, the black arrow denotes multiple nuclei per cell at the two cell stage, and the “p” refers to the paternal pronucleus in an embryo lacking a maternal pronucleus. Times in min:sec are given relative to nuclear envelope breakdown (NEBD). Scale bar, 10 µm. B. Defect maps of individual embryos observed during time-lapse recordings: embryos are listed on the left and phenotypes are listed on the top: 1; normal polar body size, 2; normal pronuclear number, 3; one nucleus per cell at two cell stage. In the long upshifts, hermaphrodites were transferred to the restrictive temperature for 5–8 hours. In the short upshifts, embryos were harvested from hermaphrodites grown at the restrictive temperature for 30 minutes. C. Amino acid alteration in the mutants. Asterisk indicates the changed residue. Homologous proteins are aligned below the *C. elegans* protein.

The meiotic spindles in the oocytes of most animals are smaller than mitotic spindles. In *C. elegans*, meiosis I and II spindles are about 8-fold smaller than the first embryonic mitotic spindle and are acentriolar. The length of microtubules during *C. elegans* meiosis are controlled in part by a katanin, a heterodimeric protein complex composed of MEI-1 and MEI-2 [43], [44]. *mei-1* encodes the AAA ATPase-containing catalytic subunit and *mei-2* encodes the targeting subunit [45]. Katanin is widely conserved and functions to shorten microtubules by severing them [46]. We isolated two recessive mutants in *mei-1*, *or642 *ts and *or646 *ts. The new *mei-1* mutants disrupt female meiotic spindle function and produce one-cell stage embryos with large misshapen polar bodies and a variable number of maternal pronuclei (range = 0–9; see Fig. 5A, B). Similar phenotypes have been described previously for other alleles [47], and both *mei-1(or642 *ts*)* and *mei-1(or646 *ts*)* failed to complement the previously identified allele *mei-1(b284)* (data not shown). We also observed multiple nuclei per two-cell blastomere, indicating chromosome segregation anomalies (which were possibly indirectly due to meiotic spindle defects), as well as occasional NCC rotation defects and transverse P_0_ spindles. The *mei-1(or642 *ts*)* and *mei-1(646 *ts*)* alleles each contain the same mutation even though they were isolated from different mutagenized nematode populations. The mutation in each changes a highly conserved lysine to glutamine at codon 202 and causes fast-inactivation of MEI-1 function (Fig. 5B, C, and Table 3). We did not find any role for *mei-1* during development after embryogenesis (Table 4).

### *spd-2* mutants

**Figure 6:**
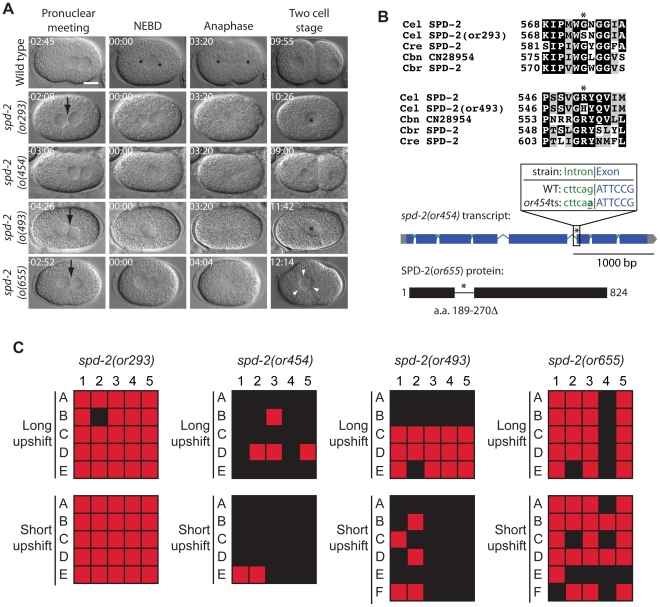
*spd-2* mutants. A. DIC time-lapse images of wild-type, *spd-2(or293 *ts*)*, *spd-2(or454 *ts*)*, *spd-2(or493 *ts*)*, and *spd-2(or655 *ts*)* embryos. In the *spd-2* mutants the pronuclei often met in the center, NCC rotation failed, a bipolar spindle failed to assemble, cytokinesis failed, and there were aberrent numbers of nuclei present at the two cell stage. The *spd-2(or293 *ts*), spd-2(or454 *ts*), and spd-2(or493 *ts*)* embryos were obtained from hermaphrodites shifted to the restrictive temperature for 5–6 hours. The *spd-2(or655 *ts*)* embryo was obtained from a hermaphrodite shifted to the restrictive temperature for ∼1 min prior to imaging. Black arrows indicate instances when pronuclei meet in the center of the embryo, asterisks represent one nucleus present in a two cell stage equivalent embryo, and white arrowheads indicate multiple nuclei. Times in min:sec are given relative to nuclear envelope breakdown (NEBD). Scale bar, 10 µm. B. Sequence alterations in the mutants. Asterisks indicates the changed residues (or nucleotide for *spd-2(or454 *ts*)*. Homologous proteins are aligned below the *C. elegans* protein. C. Defect maps for the *spd-2* mutants. Individual embryos observed during time-lapse recordings: embryos are listed on the left and phenotypes are listed on the top: 1; nuclear centrosomal complex centration, 2; nuclear centrosomal complex rotation, 3; bipolar spindle, 4; successful cytokinesis, 5; one nucleus per cell at two cell stage. In the long upshifts, hermaphrodites were transferred to the restrictive temperature for 5–8 hours. In the short upshifts, embryos were harvested from hermaphrodites grown at 15°C and immediately mounted on agar pads for imaging, which took ∼1 min.

Centrosomes are complex structures composed of centrioles surrounded by pericentriolar material that nucleates microtubules during mitotic spindle assembly and in other contexts [48]. SPD-2 is a centrosomal component required for both centriole duplication and maturation of the pericentriolar material [49], [50], [51]. We isolated four new recessive alleles of *spd-2* that cause a variety of centrosomal and microtubule-related defects, and all four alleles failed to complement the previously identified allele *spd-2(or188 *ts*)* [49] (data not shown). In one-cell embryos, we often observed that pronuclei met in the cell center instead of toward the posterior, NCC rotation and spindle assembly were absent, cytokinesis often failed, and chromosome segregation was defective (Fig. 6A). One of the *spd-2* alleles was fast-acting, *or293 *ts, while *or493 *ts and *or454 *ts mutant embryos showed weak and only partially penetrant defects when shifted to the restrictive temperature for 1 minute (Fig. 6C and Table 3). Two of the *spd-2* mutations were single amino acid substitutions: *spd-2(or293 *ts*)* changes a glycine at position 573 with a serine while *spd-2(or493 *ts*)* replaces an arginine at position 551 with a histidine (Fig. 6B and Table 2). *spd-2(or454 *ts*)* had a mutation in the last nucleotide of the fifth intron: position 2175 of the unspliced transcript was changed from a guanine to an adenine (Fig. 6B and Table 2), and this mutant produced a large percentage of viable embryos at the nonpermissive temperature (Table 1). The *spd-2* protein encoded by *spd-2(or655 *ts*)* contains an in-frame deletion that removes amino acids 189–270, and this mutant produces only 58% viable embryos at 15°C. *spd-2(or293 *ts*)* also resulted in sterile hermaphrodites with protruding vulvas when raised to adulthood at the restrictive temperature, and L1-upshifted *spd-2(or493 *ts*)* worms produced small broods (Table 4).

### *zyg-1* mutants

**Figure 7:**
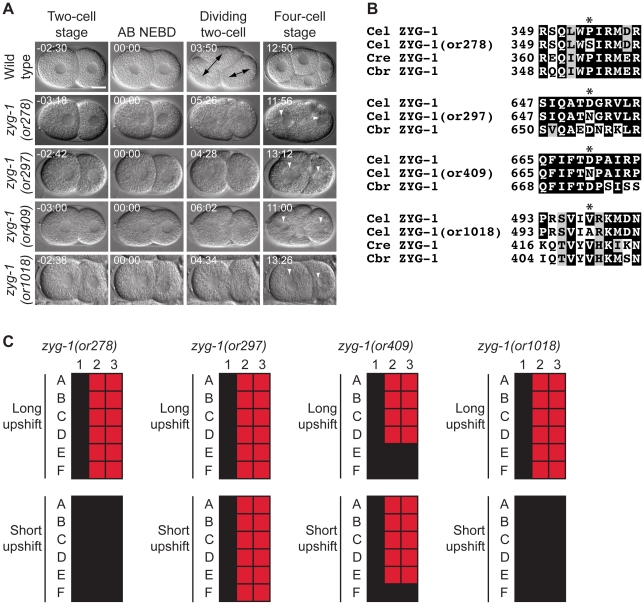
*zyg-1* mutants. A. DIC time-lapse images of wild-type, *zyg-1(or278 *ts*)*, *zyg-1(or297 *ts*)*, *zyg-1(or409 *ts*)*, and *zyg-1(or1018 *ts*)* embryos. In the *zyg-1* mutants the two cell stage blastomeres assembled monopolar spindles, cytokinesis failed, and there were multiple nuclei present at the four cell equivilent stage. The *zyg-1(or278 *ts*)*, *zyg-1(or409 *ts*)*, and *zyg-1(or1018 *ts*)* embryos were obtained from hermaphrodites shifted to the restrictive temperature for 5–6 hours. The *zyg-1(or297 *ts*)* embryo was obtained from a hermaphrodite shifted to the restrictive temperature for 30 minutes prior to imaging. Black arrows indicate normal bipolar spindles in the wild-type embryo and white arrowheads indicate multiple nuclei present at the four cell equivalent stage. Times in min:sec are given relative to AB nuclear envelope breakdown (NEBD). Scale bar, 10 µm. B. Amino acid alterations in the mutants. Asterisks indicate the changed residues. Homologous proteins are aligned below the *C. elegans* protein. C. Defect maps for the *zyg-1* mutants.Individual embryos observed during time-lapse recordings: embryos are listed on the left and phenotypes are listed on the top: 1; normal two cell embryo, 2; bipolar spindles at two cell stage, 3; one nucleus per cell at four cell stage. In the long upshifts, hermaphrodites were transferred to the restrictive temperature for 5–8 hours. In the short upshifts, embryos were harvested from hermaphrodites grown at the restrictive temperature for 30 minutes.

ZYG-1 is a polo-related kinase homologous to vertebrate SAK/PLK4 [52] that localizes to centrioles and is required for centriole duplication [53]. During fertilization, a single sperm cell provides two centrioles as well as paternal DNA to embryos, and thus embryos lacking maternal *zyg-1* function successfully proceed to the two-cell stage. However, during the AB and P_1_ cell divisions, *zyg-1* mutants form monopolar spindles because the mutant maternal ZYG-1 protein is incapable of supporting centriole duplication [53]. We isolated four new recessive alleles of *zyg-1* that cause monopolar spindles and failed mitosis in the AB and P_1_ cells (Fig. 7A and Table 1). Each of the *zyg-1* alleles alters amino acids in the C-terminal domain that appears to be nematode-specific: *zyg-1(or278 *ts*)* changes a proline to a serine at codon 354, *zyg-1(or297 *ts*)* changes an aspartic acid to asparagine at codon 652, zyg*-1(or409 *ts*)* changes a aspartic acid to an asparagine at codon 670, and *zyg-1(or1018 *ts*)* changes a valine to an alanine at codon 498 (Fig. 7B and Table 2). *zyg-1(or297 *ts*)* and *zyg-1(or409 *ts*)* appeared to be fast-acting as a 30 minute upshift resulted in penetrant defects (Fig. 7C). With the exception of *zyg-1(or297 *ts*)*, each of the new *zyg-1* TS mutants produced small broods when shifted to the restrictive temperature at the L1 larval stage (Table 4).

### *A plk*-1 mutant

**Figure 8:**
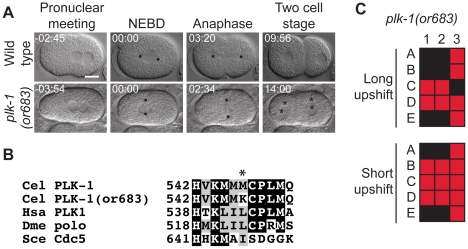
A *plk-1* mutant. A. DIC time-lapse images of wild-type and *plk-1(or683 *ts*)* embryos. In the *plk-1* mutant the nuclear centrosomal complex (NCC) failed to rotate, a transverse P_0_ spindle assembled, and the daughter blastomeres were binucleate. The *plk-1(or683 *ts*)* embryo was obtained from a hermaphrodite shifted to the restrictive temperature for 6 hours prior to imaging. Black dots represent centrosomes/spindle poles and asterisks denote multiple nuclei per cell at the two cell stage. Times in min:sec are given relative to NEBD. Scale bar, 10 µm. B. Amino acid alteration in the mutant. Asterisk indicates changed residue. Homologous proteins are aligned below the *C. elegans* protein. C. Defect map for individual embryos observed during time-lapse recordings, embryos are listed on the left and phenotypes are listed on the top: 1; nuclear centrosomal complex rotation, 2; spindle alignment, 3; one nucleus per cell at two cell stage. In the long upshifts, hermaphrodites were transferred to the restrictive temperature for 5–8 hours. In the short upshifts, embryos were harvested from hermaphrodites grown at 15°C and immediately mounted on agar pads for imaging, which took ∼1 min.

PLK-1 is a polo-like kinase that is required for meiotic spindle function, nuclear envelope breakdown, embryonic polarity, and asynchronous cell divisions in the two-cell embryo [54], [55], [56], [57]. For some of these functions, *plk-1* appears to be partially redundant with *plk-2* [56]. We isolated one new recessive allele of *plk-1, or683 ts*, which appears to only partially reduce gene function as the most penetrant phenotypes we observed at the restrictive temperature were mis-oriented (transverse) P_0_ spindles and binucleate cells at the two-cell stage (Figure 8). The *plk-1(or683 ts)* allele failed to complement the non-conditional sterile deletion allele *plk-1(ok1787)* (data not shown). Otherwise, meiotic and mitotic spindle function appeared normal. We observed penetrant defects in *plk-1* embryos after short (∼1 minute) upshifts, but as the strain produces 38% inviable embryos at the permissive temperature, we cannot conclude that it is fast-acting (Tables 1 and 3). The *plk-1(or698 ts)* mutation changes a methionine to a lysine at codon 547 that is invariably hydrophobic in various organisms (Fig. 8B). Shifting L1 larvae to the restrictive temperature resulted in sterile worms with protruding vulvae (Table 4). Interestingly, no other *plk-1* alleles have been reported (Table 5).

**Table 5:** Summary of the TS mutant loci and comparison of previously available alleles.

Locus	Allele(s) reported in this paper	Previous allele(s) published^1^	Previous TS allele(s) available^1^
*dnc-1*	*or404, or676*	yes	yes
*dnc-4*	*or618, or633*	no	no
*lit-1*	*or393*	yes	yes
*mei-1*	*or642, or646*	yes	yes
*mex-1*	*or286*	yes	no
*par-2*	*or373, or539, or640*	yes	yes
*plk-1*	*or683*	no	no
*rsa-1*	*or598*	yes	no
*spd-2*	*or293, or493, or454, or655*	yes	yes
*sur-6*	*or550*	yes	no
*tba-1*	*or594*	yes	yes
*tbb-2*	*or600*	yes	yes
*zyg-1*	*or278, or297, or409, or1018*	yes	yes

1 Information obtained from: http://www.wormbase.org.

### *par-2* mutants

**Figure 9:**
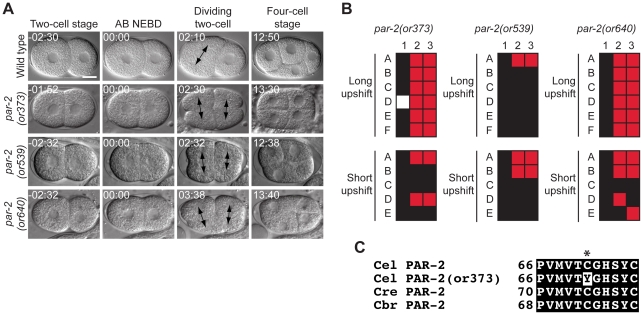
*par-2* mutants. A. DIC time-lapse images of wild-type *par-2(or373 *ts*)*, *par-2(or539 *ts*)*, and *par-2(or640 *ts*)* embryos. The blastomeres in the *par-2* mutants were of similar size at the two cell stage and initiated mitosis simultaneously, in contrast to the wild type. The *par-2(or373 *ts*)* embryo was obtained from a hermaphrodite shifted to the restrictive temperature for 5 hours prior to imaging. The *par-2(or539 *ts*)* and par-*2(or540 *ts*)* embryos were obtained from hermaphrodites shifted to the restrictive temperature for 30 minutes prior to imaging. Arrows indicate mitotic spindles at the two cell stage. Times in min:sec are given relative to AB NEBD. Scale bar, 10 µm. B. Defect map for individual embryos observed during time-lapse recordings, embryos are listed on the left and phenotypes are listed on the top: 1; Normal one cell embryo; 2; assymetric two cell embyro, 3; asynchronous two cell divisions. In the long upshifts, hermaphrodites were transferred to the restrictive temperature for 5–8 hours. In the short upshifts, embryos were harvested from hermaphrodites transferred to the restrictive temperature for 30 minutes. C. Amino acid alteration in the *par-2(or373 *ts*)* mutant. Asterisk indicates the changed residue. Homologous proteins are aligned below the *C. elegans* protein.

*par-2* is required for anterior-posterior polarity in the one-cell zygote and encodes a RING finger protein [58], [59], [60]. We isolated three new recessive *par-2* mutants that disrupt zygote polarity; all three alleles failed to complement the previously identified allele *par-2(lw32)* (data not shown). In two-cell embryos the lack of polarity was revealed by blastomeres having equal size that entered mitosis at the same time, in contrast to wild-type embryos that display asymmetric AB and P_1_ cell sizes and timing of mitotic entry (Fig. 9A). *par-2(or539 ts)* had low penetrance cellular defects after both short and long upshifts, consistent with the fact that it produces a high percentage of viable embryos at the restrictive temperature (Table 1). All of the alleles appeared to be fast-acting, although for *par-2(or373 ts)* and *par-2(or640 ts)* the penetrance of defects observed after short upshifts was lower than seen after long upshifts (Fig. 9B, Table 3). We found that *par-2(or373 ts)* contained a cysteine to tyrosine change at codon 71 (Fig. 9C and Table 2). Finally, *par-2(or373 ts)* and *par-2(or539 ts)* worms were fertile and produced inviable embryos when grown to adulthood from the L1 larval stage, but the *par-2(or640 ts)* mutant worms were sterile after L1 temperature upshifts (Table 4).

### *lit-1* and *mex-1* mutants

**Figure 10:**
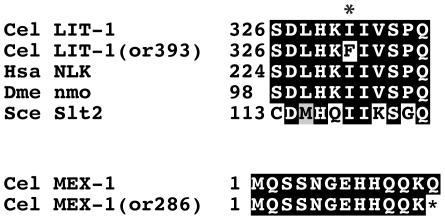
Sequence alterations in the *lit-1(or393 *ts*)* and *mex-1(or286 *ts*)* mutants. Amino acid alterations in the mutants. Asterisks indicates changed residues. Homologous proteins are aligned below the *C. elegans* protein for LIT-1. A glutamine codon was changed to a stop codon in the *mex-1(or286 *ts*)* allele.

*lit-1* and *mex-1* control embryonic cell fate patterning. LIT-1 is a kinase that controls anterior/posterior daughter cell fates beginning at the 6-cell stage when the ventral-most embryonic cell called EMS divides along the anterior/posterior body axis [61], [62], [63]. MEX-1 is a zinc finger protein that restricts blastomere identity at the 8-cell stage but also has been shown to affect anterior-posterior polarity at the one-cell stage [64], [65], [66]. We found one new *lit-1* mutant, *or393 ts* and one new *mex-1* mutant, *or286 ts*, which failed to complement the previously identified alleles *lit-1(or131 ts)* and *mex-1(zu120)*, respectively (data not shown). *lit-1(or393 ts)* hermaphrodites produced embryos that contained fewer intestinal cells, as compared to wild-type worms (not shown). *lit-1(or393 ts)* was recessive (Table 1) and did not exhibit any phenotypes other than embryonic lethality when grown to adulthood at the restrictive temperature from the L1 larval stage (Table 4). We found that codon 331 was changed from an isoleucine to a phenylalanine in *lit-1(or393 ts)* strain (Fig. 10 and Table 2). *mex-1(or286 ts)* hermaphrodites generated embryos that, as reported previously for other alleles, produced a large excess of pharyngeal tissue (data not shown). L1 upshift experiments revealed that *mex-1* worms produced either small broods or were sterile (Table 4). The sequence alteration in the *mex-1(or286 ts)* mutant changed a glutamine at codon 13 to a stop codon (Fig. 10 and Table 2).

## Discussion

Most of the effort to investigate essential *C. elegans genes* has thus far focused on gene products that, when defective, exhibit early embryonic cell division defects. However, extending efforts to investigate previously ignored or poorly studied mutant classes is now more appealing with the rapid cloning methods available. These mutant classes include eggshell-defective mutants, sterile or small brood-producing mutants, mutants with delayed progression through S phase, and mutants with normal early embryonic cell divisions but highly penetrant lethality presumably due to defects later in embryogenesis. By first identifying what genes are affected in such mutants, research effort might be more productively focused on conserved genes with important roles in other model systems and in human health.

Most of the mutants we describe here have been studied previously, either by using mutant alleles or RNAi depletion, and the cellular phenotypes we present mirror what has been presented previously. However, these new strains should still prove valuable for the phenotypic analysis of embryos and worms after bypassing the earliest defects, by providing sensitized backgrounds for use in modifier screens, for defining temperature-sensitive periods, and as templates for engineering TS alleles in homologous genes (see below). One interesting finding we have made is that fast-acting TS alleles (as defined by our criteria) are not unusual (Table 3). Thirteen of the alleles we have characterized here are potentially fast-acting, and we could not make a determination on six others because of either 1) weak or low penetrance defects or, 2) high lethality at the permissive temperature. In fact, only three mutations were definitively not fast-acting. In future assays, it may be useful to grow worms and conduct rapid upshifts in a room maintained at 15°C by use of a temperature-controlled microscope stage, in order to bypass mounting embryos at room temperature (which would likely allow one to further clarify the “Unclear” determinations in Table 4). The observation that∼50% of the conditional mutants we analyzed are potentially fast-acting provides additional incentive to isolate more TS alleles.

Of the 13 loci we have described, no genetic alleles have been described for two *(dnc-4 and plk-1)*, while no TS alleles have been described for three others *(mex-1, rsa-1, and sur-6;* see Table 5). Sixteen of the sequenced TS alleles are single mis-sense mutations and another allele, *dnc-1(or676 ts)* has two mis-sense mutations. Two of the TS alleles, *dnc-4(or633 ts)* and *spd-2(or454 ts)*, change nucleotides in introns that likely affect RNA splicing, and interestingly, *dnc-4(or633 ts)* is highly temperature-sensitive while *spd-2(or454 ts)* is less so (Table 1). It would be interesting to test if engineering either of these splice site mutations into other genes would also confer conditional gene function. One of the mutants contains an in-frame deletion, *spd-2(or655 ts),* while *mex-1(or286 ts)* has a premature stop codon, and both of these mutants produce a substantial fraction of inviable embryos at the permissive temperature (Table 1). As 81% of our TS alleles were mis-sense mutations, searching for mis-sense mutations in mutant genome exon sequences should lead to finding the causative mutations in most TS mutants. Finally, the amino acid substitutions in nine of the alleles alter residues that are similar (seven of them are identical) in homologous proteins in vertebrates, *Drosophila melanogaster*, and budding yeast. Thus, it may be possible to engineer these changes in other organisms to obtain TS alleles. Eight of the TS alleles alter residues only conserved within nematodes. As a substantial fraction of the TS mutations we have described either do affect widely conserved residues, or are not fast-acting, further efforts to identify additional conditional mutations in even these essential *C. elegans* genes may prove valuable, and TS mutations have yet to be identified for most of the roughly 2500 essential genes present in the *C. elegans* genome.

## Materials and Methods

### *C. elegans* strains and culture

Strains were grown under standard laboratory conditions [67]. The temperature sensitive mutants were maintained in a 15°C incubator and shifted to a 26°C incubator to perform temperature upshifts for determining embryonic lethality. Mutants were isolated in a *lin-2(e1309)* background, as previously described [25]. For performing embryonic viability counts, we transferred >10 L4 hermaphrodites to individual plates and grew them at the permissive (15°C) or restrictive (26°C) temperatures until broods were produced. We then removed the worms and allowed the embryos to develop prior to counting viable and inviable progeny. For testing embryonic lethality in heterozygous mothers, we crossed the mutants to a *him-5* strain and tested the F1 progeny as described above. For determining the phenotypes of the TS mutants when shifted to the restrictive temperature from the L1 larval stage, we performed hypochlorite treatments and allowed the embryos to hatch in M9 buffer at 15°C. We then plated∼100 synchronized L1 larvae onto a plate and grew them at 26°C until they reached adulthood.

### Microscopy

Imaging was performed by mounting embryos grown at either 15°C or 26°C on 3% agar pads on microscope slides and sealed with a cover slip. Mounting the embryos was performed at room temperature and usually took 1–2 minutes. Nomarski time lapse images were acquired at a frame rate of 1 image/2 seconds on Zeiss (http://www.zeiss.com) axioskop microscopes equipped with CCD cameras using ImageJ software (http://rsbweb.nih.gov/ij/). Microscopy was performed at room temperature in a room maintained at 24°C. Images were adjusted for contrast in ImageJ.

### Mutation identification

Sanger DNA sequencing was performed at the University of Oregon Genomics facility for most genes. We used PCR reactions to amplify 1–2 Kb gene fragments using *Taq* DNA polymerase (Invitrogen). The PCR reactions were run on agarose gels prior to isolating the DNA using a Qiagen QIAquick gel extraction kit. For *plk-1* and *tbb-2,* we used a procedure called interval pull down sequencing which we have developed (manuscript in preparation). Briefly, we isolated mutant genomic DNA, sheared it, annealed it to fosmids containing wild-type genomic DNA and used beads to isolate megabase regions of interest. This purified DNA was subjected to Illumina sequencing at the University of Oregon Genomics facility.

### Sequence alignments

We used Wormbase (http://www.wormbase.org/) to obtain homologous proteins encoded in the *Homo sapiens*, *Drosophila melanogaster*, and *Saccharomyces cerevisiae* genomes. In cases where homologous proteins (or homologous domains) were not present, we performed alignments with other nematode sequences. Protein sequences were aligned with default parameters in CLUSTALW (http://align.genome.jp/) and outputted as alignments using the BOXSHADE package (http://www.ch.embnet.org/software/BOX_form.html).
